# Oyster RNA-seq Data Support the Development of *Malacoherpesviridae* Genomics

**DOI:** 10.3389/fmicb.2017.01515

**Published:** 2017-08-09

**Authors:** Umberto Rosani, Paola Venier

**Affiliations:** Department of Biology, University of Padua Padua, Italy

**Keywords:** *Malacoherpesviridae*, RNA-seq, viromes, bivalve, OsHV-1, *Herpesvirales*

## Abstract

The family of double-stranded DNA (dsDNA) *Malacoherpesviridae* includes viruses able to infect marine mollusks and detrimental for worldwide aquaculture production. Due to fast-occurring mortality and a lack of permissive cell lines, the available data on the few known *Malacoherpesviridae* provide only partial support for the study of molecular virus features, life cycle, and evolutionary history. Following thorough data mining of bivalve and gastropod RNA-seq experiments, we used more than five million *Malacoherpesviridae* reads to improve the annotation of viral genomes and to characterize viral InDels, nucleotide stretches, and SNPs. Both genome and protein domain analyses confirmed the evolutionary diversification and gene uniqueness of known *Malacoherpesviridae*. However, the presence of *Malacoherpesviridae*-like sequences integrated within genomes of phylogenetically distant invertebrates indicates broad diffusion of these viruses and indicates the need for confirmatory investigations. The manifest co-occurrence of OsHV-1 genotype variants in single RNA-seq samples of *Crassostrea gigas* provide further support for the *Malacoherpesviridae* diversification. In addition to simple sequence motifs inter-punctuating viral ORFs, recombination-inducing sequences were found to be enriched in the OsHV-1 and AbHV1-AUS genomes. Finally, the highly correlated expression of most viral ORFs in multiple oyster samples is consistent with the burst of viral proteins during the lytic phase.

## Introduction

The virus family of double-stranded DNA (dsDNA) *Malacoherpesviridae* refers to only those *Herpesvirales* which affect mollusks, with the Haliotid herpesvirus, and bivalve Ostreid herpesvirus-1 being highly similar virus variants and the only family members described so far (Davison et al., 2005; Savin et al., 2010). Based on phylogenetic analysis, *Malacoherpesviridae* are distantly related to other *Herpesvirales* families, namely *alpha-, beta-, gamma*-, and *allo-herpesviridae* (Davison et al., 2009; Iranzo et al., 2016). In general, the considerable genome size of *Herpesvirales* (125–290 kb) supports complex transcriptional landscapes, including several coding (ORFs) and non-coding RNAs (ncRNAs) such as microRNAs (miRNAs). Modulation of latent vs. lytic phases guarantees long-term survival and efficient propagation of *Herpesvirales*, although viral genomes are exposed to mutational pressure during their latency state into the cell nucleus (Brown, 2014). In *alpha*- and *gamma*-*herpesvirales*, different recombination-initiating motifs can activate host genome integrity pathways like homologous recombination-dependent DNA repair (HR), a virus-protective strategy proposed as being crucial for *Herpesvirales* biology (Brown, 2017; Piekna-Przybylska et al., 2017).

Speed, sensitivity, and resolution of current high-throughput sequencing (HTS) technologies have been successfully used to unlock the transcriptional landscape of Kaposi's sarcoma-associated herpesvirus, which is characterized by alternative splicing of viral introns, polycistronic mRNAs, alternative transcription starting sites, and a significant repertoire of ncRNAs (Arias et al., 2014; Strahan et al., 2016). Although transcriptome complexity could be a general feature of *Herpesvirales* (Stern-Ginossar et al., 2012; Oláh et al., 2015; Tombácz et al., 2016), their marked host-adaptation and phylogenetic diversity discourage any oversimplification.

As reported in Table 1, the *Malacoherpesviridae* genomes of Ostreid herpesvirus type-1 (OsHV-1), *Chlamys* acute necrobiotic virus (AVNV), and Haliotid herpesvirus 1 (AbHV-1-AUS) were sequenced in 2005, 2010, and 2013, respectively. A micro-variant genome called μVar was described in 2010 by mapping its sequence differences on the OsHV-1 genome; a further variant of OsHV-1 (OsHV-1-SB) from diseased *Scapharca broughtonii* and a Taiwanese variant of AbHV-1-AUS (AbHV-1-TAI) were sequenced and recorded at NCBI in 2015 and 2016, respectively. So far, most published studies have focused on the variant μVar, a genotype associated with severe and worldwide events of *Crassostrea gigas* mortality (Segarra et al., 2010; Arzul et al., 2017). The variant μVar differs from the reference genome because of small and large deletions and due to some single nucleotide changes (Segarra et al., 2010). Viruses most likely exist as a mixture of genotypes, and also a recent analysis of OsHV-1 DNA occurring in wild oyster stocks in Italy indicated the co-occurrence of slightly different OsHV-1 genotypes (Burioli et al., 2016). Expression profiles of both *C. gigas* and OsHV-1s have been investigated by suppression subtractive hybridization (Renault et al., 2011), qPCR (Segarra et al., 2014b; Green et al., 2015a) and by dual RNA-seq applied to oysters which were experimentally infected (He et al., 2015) and naturally infected (Rosani et al., 2015). Although OsHV-1 genotypes have been mainly reported in Pacific oysters, OsHV-1 was recently associated with mortality events of the Chinese bivalve *S. broughtonii* (Renault et al., 2012; Bai et al., 2015; Xia et al., 2015), whereas other bivalve spp. might act as simple virus carriers (Burge et al., 2011) or be not susceptible (Tan et al., 2015). In the same way, the closely related abalone herpesvirus represents an important pathogen for the gastropod family of *Haliotis* spp. (Chang et al., 2005; Savin et al., 2010; Corbeil et al., 2016). Overall, the lack of stringent host-virus specificity indicates *Malacoherpesviridae* as dangerous pathogens for the entire mollusk aquaculture sector.

**Table 1:** Malacoherpesviridae genomes.

**Virus name**		**Preferred host**	**NCBI ID**	**Genome size (kb)**	**No. of annotated ORF**	**References**
Bivalve-	OsHV-1 [1]	*C. gigas*	AY509253	207	136	Davison et al., 2005
	OsHV-1-μVAR [2]	*C. gigas*	/	201	134	Segarra et al., 2010
	OsHV-1-SB [3]	*S. broughtonii*	KP412538	199	66	Xia et al., 2015
	AVNV [4]	*Chlamys* spp.	GQ153938	211	134	Ren et al., 2013
Gastropod-	AbHV-1-AUS [5]	*Abalone* spp.	NC_018874	212	118	Savin et al., 2010
	AbHV-1-TAI [6]	*Abalone* spp.	KU096999	199	74	NCBI, April 2016

*Virus name and related host, NCBI ID of the reference genome, genome size, and percentage of annotated ORF are reported for bivalve and gastropod Malacoherpesviridae. Numbering in square brackets is a reference for Table 3*.

While the analysis of infected samples has greatly advanced the general understanding of antiviral pathways in bivalve mollusks (Renault et al., 2011; Corporeau et al., 2014; Segarra et al., 2014a; Green et al., 2015b; Moreau et al., 2015; Martenot et al., 2017), the biology of *Malacoherpesviridae* is still obscure. The uniqueness of this viral family within the frame of poorly characterized marine mollusk viromes raises questions as to their evolutionary history, infection mechanisms in different hosts, and the functional roles of their proteins. In the absence of permissive cell lines, the development of “*ad hoc*” HTS approaches is today the most promising way to disclose the *Malacoherpesviridae* peculiarities.

In the present work, we used available mollusk RNA-seq data as a source of *Malacoherpesviridae* reads to perform a detailed genomic remapping. Also in comparison with the most recently sequenced virus genomes, we present and discuss the diversity and uniqueness of *Malacoherpesviridae*, taking into consideration SNPs and sequence motifs and raising some annotation incongruities. In particular, we report the identification of *Malacoherpesviridae*-like elements endogenously occurring in invertebrate genomes, and sequence motifs related to viral genome protection mechanisms and comprehensive OsHV-1 transcription data.

## Materials and methods

### Sequence data

We retrieved the whole genome sequences and classification of 2,665 dsDNA viruses, including 82 *Herpesvirales*, from the NCBI database. A total of 96 RNA-seq (Zhang et al., 2012) and 8 miRNA-seq (Xu et al., 2014) samples from *C. gigas* as well as 159 RNA-seq samples from different *Haliotis* spp. were retrieved from the SRA archive (Supplementary File 1). The *C. gigas* genome was obtained from *Ensemble Metazoa v.3* (Zhang et al., 2012). *MiRBase* release 21 was downloaded from http://www.mirbase.org/ (Kozomara and Griffiths-Jones, 2014). Additionally, scaffolds of genome drafts of Cephalochordates (*Branchiostoma floridae, Branchiostoma belcheri*, and *Asymmetron lucayanum*), *Annelida (Capitella teleta, Hydroides elegans, and Helobdella robusta)*, gastropods *(Lottia gigantea, Aplysia californica, and Conus tribblei)*, and bivalves *(Mytilus galloprovincialis, C. virginica, Mizuhopecten yessoensis, Modiolus philippinarum, and Bathymodiolus platifrons)* were retrieved from the NCBI archive and used to compose a genomic blast database (Camacho et al., 2009).

### Identification and mapping of Malacoherpesviridae reads

If not differently indicated, all the analyses were performed using CLC Genomic Workbench v.10.0 (Qiagen, Germany). RNA reads were trimmed for quality, allowing a maximum of two ambiguous bases and a quality threshold of Q20. To reduce false positive viral hits, the mapping of *C. gigas* RNA-seq reads on the oyster genome was performed with the *large gap read mapping* (LGRM) tool. Sequence reads not mapping on the *C. gigas* genome were stringently mapped (0.9 and 0.9 for length and similarity fraction, respectively) on known *Malacoherpesviridae* genomes and the resulting positive hits were labeled as “*Malacoherpesviridae* reads” and retained for subsequent analyses. Viral spliced reads were retrieved by mapping the reads on known *Malacoherpesviridae* genomes with LGRM. In the absence of *Haliotid* spp. genomes, abalone RNA reads were directly mapped on known *Malacoherpesviridae* genomes with stringent mapping parameters.

### Sequence alignment and phylogenetic analysis

*Malacoherpesviridae* genomes were aligned with the progressive Mauve algorithm included in the MAUVE tool (Darling et al., 2010). *Malacoherpesviridae* ORFs were compared with other *Herpesvirales* ORFs extracted from the downloaded genomes. Predicted proteins were aligned with MUSCLE v.3.8 (Edgar, 2004) and phylogenetic trees were generated using the Neighbor Joining algorithm and UPGMA algorithms, with Jukes-Cantor distance estimation and applying 1,000 bootstrap replicates with a significance cut-off set at 500. *Blast* searches were performed locally using BLAST+ (Camacho et al., 2009), whereas conserved domains as well as peptide and transmembrane regions were identified by using InterProScan v.60, SignalP, and THMM tools, respectively (Petersen et al., 2011). Putative *Malacoherpesviridae* endogenous viral elements (EVEs) were searched on 15 invertebrate genomes using *tblastn* with a cut-off *E*-value of 10^−50^ with all the OsHV-1 ORFs as query. The resulting hits were extracted and further inspected.

### Prediction of miRNA precursors and recombination-initiating motifs

Two *ab-initio* tools for the detection of miRNA precursors were tested on *Malacoherpesviridae* genomes, namely miRPara (Wu et al., 2011) and VMir (Grundhoff et al., 2006). Briefly, miRPAra is a Support Vector Machine tool that provides 76 parameters predictive of putative hairpin regions on the basis of experimentally verified animal, plant, and virus miRNA models. VMir slides a sequence window of adjustable size across the viral genomes and then employs the RNA fold algorithm to predict structures with minimal free energy folding. Pre-miRNA candidates were identified and scored by evaluating the structural features of known pre-miRNA hairpins. Results obtained with miRPAra and VMir were compared and only common predicted structures were retained as putative *Malacoherpesviridae* miRNAs. To ascertain the presence of any viral miRNAs among *C. gigas* miRNA-seq reads, *de-novo* assembled consensus sequences generated from the 8 miRNA-seq samples were mapped on both *C. gigas* and *Malacoherpesviridae* genomes.

Nucleotidic motifs indicative of polyadenylation sites (PAS) or representative of conserved patterns were identified by simple textual searches along whole *Malacoherpesviridae* genomes or by applying the MEME tool (Bailey and Elkan, 1994) in the 3′ and 5′UTR ORF regions (the latter were defined as the 100 nt before the starting codon and after the stop codon, respectively). All viral dsDNA genomes were scanned for the presence of six recombination-initiating motifs possibly activating homologous recombination-dependent DNA repair (host HR), and enrichment ratios were calculated as reported in (Brown, 2014). Briefly, sequence motifs were identified in both original sequences and randomized sequences obtained with *shuffleseq* (default parameters, EMBOSS Explorer, http://cys.genomics.purdue.edu/emboss/), and then counted. The amount of a given sequence motif in each viral genome was normalized by genome length before computing the motif enrichment (ratio higher than two between original and randomized sequences).

### OsHV-1 expression analysis and SNP calling

To quantify the expression of viral ORFs, all *Malacoherpesviridae* reads were stringently mapped on the OsHV-1 reference genome (GenBank ID: AY509253) setting both length and similarity parameters to 0.9. Starting from the read counts, Transcripts Per Million (TPM) values were computed according to Wagner et al. (2013) in order to evaluate viral expression patterns by Principal Component Analysis (PCA) and ORF clustering (Euclidean distance, single linkage).

Single Nucleotide Polymorphism (SNP) analysis was performed on mapping files. Nucleotide changes were called “SNP” if present in at least 5% of the locally aligned reads using the following parameters: minimum average quality of the five surrounding bases, 15; minimum quality of central base, 20; minimum required coverage, 100x. Subsequently, SNP analysis was repeated on read mappings joined by sample origin and SNPs were compared among groups. SNP calling parameters were maintained except for the coverage, which was lowered to 20x to account for a smaller number of aligned reads.

## Results

### Tracing the phylogenetic history of divergent *Herpesvirales* (*Malacoherpesviridae*)

**Figure 1:**
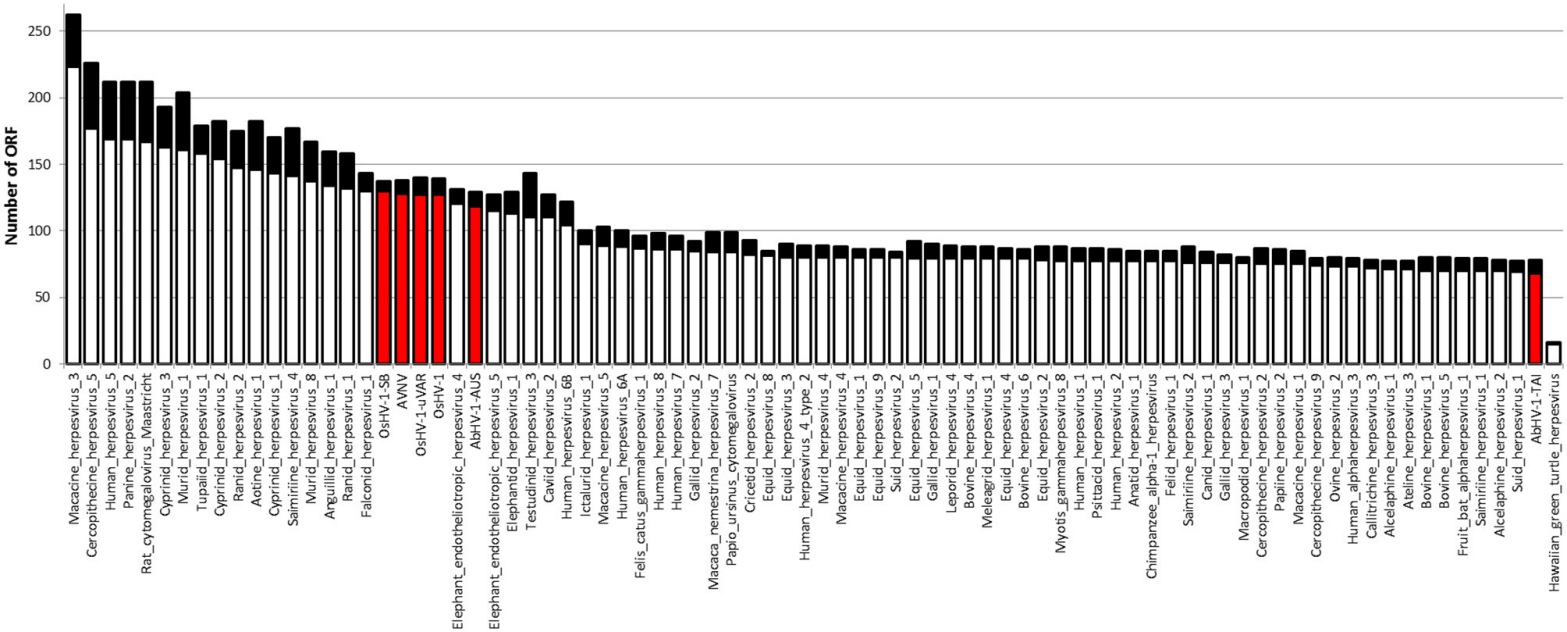
Number of ORFs identified in *Herpesvirales* genomes. The black part of each bar indicates the ORFs fraction with a predicted signal peptide. *Malacoherpesviridae* genomes are highlighted by the red bars.

*Malacoherpesviridae* display a genome size of 199–212 kb and a number of predicted ORFs ranging from 66 for AbHV-1-TAI to 136 for OsHV-1, always covering most of the genome sequence (Table 1). As for other *Herpesvirales*, 5–15% of the mollusk viral ORFs have a signal peptide region (Figure 1). Multiple alignment of whole *Malacoherpesviridae* genomes highlighted conserved sequence blocks clearly discriminating two genome types, namely bivalve and gastropod viruses, with very few regions of high similarity between them (e.g., *ribonucleoside-diphosphate reductase*, light violet blocks) and other genomic regions shared between two only of the three bivalve *Malacoherpesviridae* genomes (red and light green boxes, Supplementary File 2). These regions refer either to intergenic segments, like the variable microsatellite regions that differentiate OsHV-1 from the μVar variant (Segarra et al., 2010; Martenot et al., 2013), or to deletions/insertions that modify the coding potential (e.g., the large deletion of ORF36-37 in the μVar variant (Renault et al., 2012). Among the latter discriminant features, we remarked a 2.7 kb insertion characterizing the AVNV and OsHV-1-SB genomes (position 60–63 kb) and encoding three ORFs with unknown function (OsHV-1-SB ORF125, a putative secreted protein; ORF126 and ORF127, a putative transmembrane protein). We later exploited these virus-specific regions to determine which type of virus variant was present in a given RNA sample.

**Figure 2:**
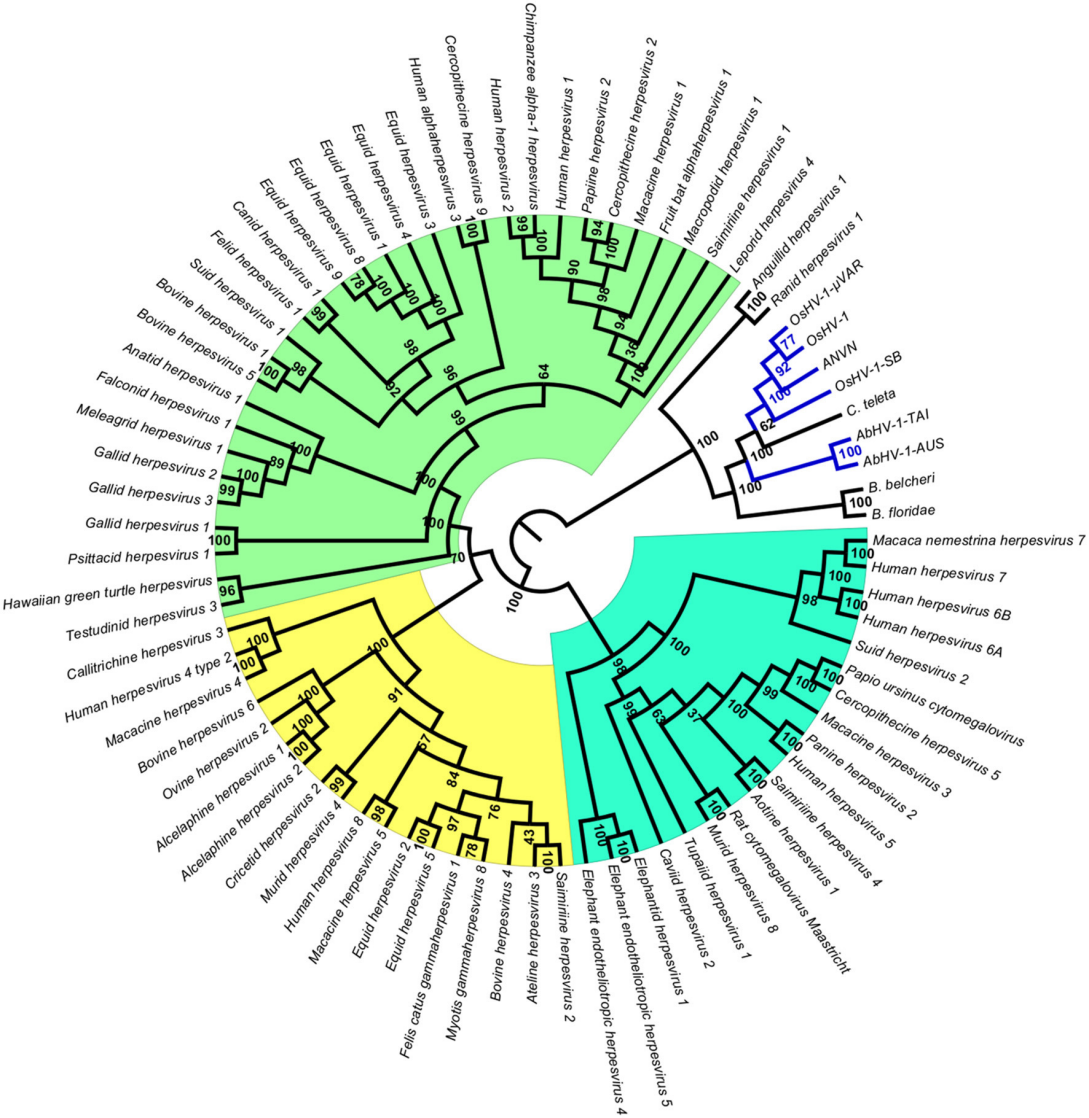
Phylogenetic tree of *catalytic subunit of DNA polymerase*. The blue lines represent *Malacoherpesviridae* hits. Background colors highlight *alpha-Herpesvirales* in green, *beta-Herpesvirales* in blue, and *gamma-Herpesvirales* in yellow. The circular cladogram was computed with the Neighbor Joining algorithm and Jukes-Cantor distance estimation. Bootstrap values are reported for each node as percentages calculated over 1,000 performed replicates, with a significance cutoff set at 500.

To obtain data useful for reconstructing the evolutionary history of *Malacoherpesviridae*, we searched for EVEs in a number of invertebrate genomes, including nine bivalve and gastropod genome drafts. The analyzed invertebrate genomes did not include any putative *Malacoherpesviridae* EVEs, the sole exception being ORFs denoting a *Ribonucleotide reductase big subunit*, found in the *C. gigas, M. philippinarum, C. tribbei*, and *C. teleta* genomes. As reported for *B. floridae* (Savin et al., 2010), we also identified a portion of a genome scaffold showing high similarity with Herpesvirales sequences in lancelet (Branchiostoma) spp. and in annelid Capitella teleta. These scaffolds matched several *Herpesvirales* ORFs, including viral DNA polymerase (Table 2). Phylogenetic trees based on the *catalytic subunit of DNA polymerase* and generated with two different algorithms always showed well-supported clades for the alpha-, beta-, and gamma-Herpesviridae, and for a clade containing *Malacoherpesviridae, allo-Herpesvirales*, and three DNA polymerase sequences retrieved from invertebrate genomes (Figure 2). In detail, the annelid sequence clustered with bivalve *Malacoherpesviridae*, whereas the two lancelet sequences clustered as an out group that was more similar to abalone *Malacoherpesviridae*. These results further emphasize the evolutionary divergence of known mollusk viruses from other *Herpesvirales* as reported by Davison et al. (2005) and Iranzo et al. (2016). Moreover, the presence of *Herpesvirales* EVEs very similar to *Malacoherpesviridae* (*Malacoherpesviridae*-like) is confirmed not only in *B. floridae* (Savin et al., 2010) but also in the *B. belcheri* and *C. teleta* genomes. Depending on the applied algorithm, allo-Herpesvirales clustered as an outgroup of *Malacoherpesviridae* or of other *Herpesvirales* (data not shown).

**Table 2:** Identification of *Malacoherpesviridae* endogenous viral elements *(*EVEs) in invertebrate genomes.

***Malacoherpesviridae* ORF**	**Blast hit**	**E-value (10^E^)**	**Scaffold ID**
*DNA polymerase*	*C. teleta*	−*172*	Scaffold_559
	*B. floridae*	−*104*	ABEP02031171
	*B. belcheri*	−*98*	FQTN01000253
*DNA packaging terminase*	*C. teleta*	−*105*	Scaffold_559
	*B. floridae*	−*99*	ABEP02031173
	*B. belcheri*	−*95*	FQTN01000253
*ORF54*	*B. floridae*	−*98*	ABEP02031173
	*B. belcheri*	−*91*	FQTN01000253
	*C. teleta*	−*53*	Scaffold_559
*Ribonucleotide reductase big subunit*	*C.gigas*	−*97*	C36000
	*C. tribbei*	−*65*	
	*M. philippinarum*	−*63*	Scaf_11254
	*C. teleta*	−*61*	Scaffold_559
*ORF68-secreted protein*	*B. floridae*	−*96*	ABEP02031173
	*B. belcheri*	−*92*	FQTN01000253
	*C. teleta*	−*67*	Scaffold_559
*ORF47*	*B. floridae*	−*74*	ABEP02031173
	*C. teleta*	−*59*	Scaffold_559
*DNA primase*	*C. teleta*	−*67*	Scaffold_559
	*B. floridae*	−*62*	ABEP02031171
	*B. belcheri*	−*61*	FQTN01000253

### *Malacoherpesviridae*-specific genes support the divergence from other *Herpesvirales*

**Figure 3:**
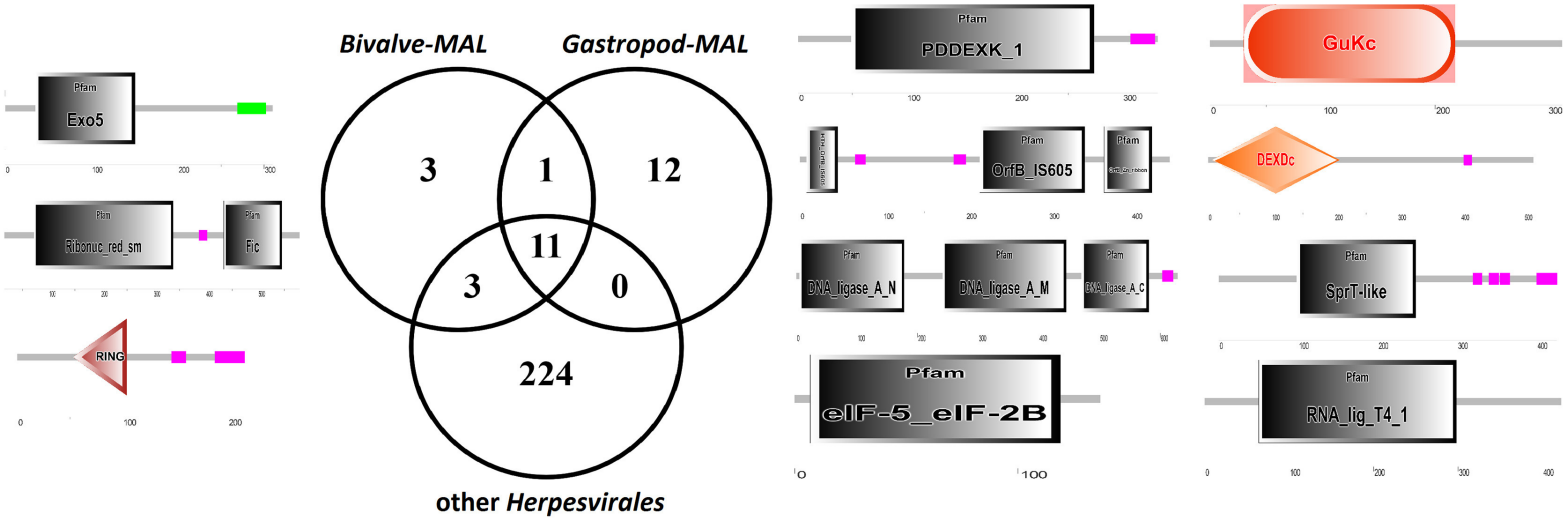
Venn diagram of the protein domains detected in bivalve *Malacoherpesviridae*, gastropod *Malacoherpesviridae*, or other *Herpesvirales*. Organization of protein domains (with PFAM IDs) of bivalve-exclusive **(left)** and gastropod-exclusive **(right)** viral proteins.

The uniqueness of most of the *Malacoherpesviridae* genes hampers a comparative identification of conserved protein domains and homologous genes in public sequence databases. Moreover, *Malacoherpesviridae* share amongst themselves, and with other *Herpesvirales*, a small number of protein domains which mainly pertain to transcription-related proteins. As previously reported by Davison et al. (2005) for OsHV-1, the BIR domain (PF00653) was the only one exclusively found in all *Malacoherpesviridae*, whereas some other protein domains were identified as specific to bivalve or gastropod herpesviruses (Supplementary File 3). Although some *Herpesvirales* possess proteins inhibiting the host apoptotic pathways, e.g., proteins with a partial BIR domain or a *Bcl-2-like* protein (Wang et al., 2002; Gallo et al., 2017), a highly-confident BIR domain was uniquely found in *Malacoherpesviridae*, whereas the FIC, Exo5, and zf-RING_5 domains were detected only in bivalve *Malacoherpesviridae*. FIC (PF02661) characterizes proteins mediating post-translational modifications (Roy and Cherfils, 2015) and Exo5 characterizes the same clan of other herpesvirus exonucleases (Herpes_UL24 and Herpes_alk_exo domains) including the gastropod *Malacoherpesviridae* exonuclease (PDDEXK_1 domain). Through stable coordination of Zn cations, zf-RING_5 fingers acquire different binding specificities for DNA, RNA, proteins, and/or lipid targets, and therefore pleiotropic roles. For instance, the RING domain of the *immediate-early protein* (ICPO) of herpes simplex virus 1 (HSV-1) has ubiquitin ligase activity, enabling protein targeting for degradation, and inhibits interferon-stimulated host gene production (Taylor et al., 2014). As reported in Figure 3 and Table 3, the higher number of protein domains unique to gastropods and related to bivalve *Malacoherpesviridae* (12 and 3, respectively) is mainly due to a couple of viral genes including six protein domains and encoding a *DNA ligase* (present on both AbHV-1-AUS and AbHV-1-TAI genomes) and a *methyltransferase-like* (present uniquely in AbHV-1-AUS).

**Table 3:** Viral ORFs with *Malacoherpesviridae*-specific protein domains.

**ORF annotation**	**Virus ID**	**Domain ID**	**No. of MAL gene**	**Domain occurrence**	
				**Other viruses**	**Mollusk genomes**
Apoptosis inhibitor	1, 2, 3, 4, 5, 6	BIR	18 (6)	dsDNA and RNA viruses	Yes
Ribonucleotide reductase, s.s.	1, 2, 3, 4	Ribonuc_red_sm, Fic	4 (1)	*Caudovirales, Nimaviridae, Nudiviridae*	Yes
Guanylate kinase	5, 6	Guanylate_kin	2 (1)	Poxviridae, *Caudovirales*	Yes
DNA ligase	5, 6	DNA_ligase_A_M, DNA_ligase_A_N, DNA_ligase_A_C	2 (1)	dsDNA viruses	Yes
Eukaryotic translation initiation factor-5	5	eIF-5_eIF-2B	1 (1)	*Baculoviridae, Marseilleviridae*	Yes
Exonuclease	1, 2, 3, 4	EXO5	4 (1)	/	Low similarity
Exonuclease	5, 6	PDDEXK_1	2 (1)	dsDNA viruses, *Caudovirales*	Low similarity
/	5, 6	SprT-like	2 (1)	*Caudovirales, Baculoviridae*	Low similarity
RNA helicase	5, 6	ResIII	2 (1)	dsDNS and ssRNA viruses	Low similarity
Zinc finger	1, 2, 3, 4	zf-RING_5	4 (1)	*Mimiviridae*	Low similarity
RNA ligase	5, 6^*^	RNA_lig_T4_1	2 (1)	*Caudovirales, Baculoviridae*	No
Methyltransferase/	5	OrfB_Zn_ribbon,	1 (1)	*Caudovirales Mimiviridae Phycodnaviridae*	No
transposase		OrfB_IS605,			
		HTH_OrfB_IS605			

*From left to right: putative ORF annotation and Malacoherpesviridae family members in which they are present (see numbering in Table 1), domain description, total number of Malacoherpesviridae genes showing the specified domain (in parentheses, the number of unique genes), and occurrence of the same domain in other viral families as well as in mollusk genomes*.

* *Partial or incomplete sequences are also present in bivalve Malacoherpesviridae*.

### Putative protein counterparts of *Malacoherpesviridae*-specific domains

We further investigated the putative origin of viral genes including *Malacoherpesviridae-exclusive* (among *Herpesvirales*) protein domains by domain-based searches of similar genes in public databases (NCBI) and in available mollusk genomes. Accordingly, we were able to assign *Malacoherpesviridae*-specific domains to 17 viral genes, present in all or some of the known *Malacoherpesviridae*. Eleven of them matched mollusk counterparts with similar domain organization (*blastp* similarity value < 10^−5^). Searches within the *C. gigas* gene models identified genes characterized by BIR, guanylate kinase, DNA ligase, FIC, and *eukaryotic translation initiation factor* domains. Other *Malacoherpesviridae*-specific domains showed scarce similarity with host genes characterized by EXO5, PDDEXK_1, zf-RING_5 and Sprt-like domains, whereas the viral RNA-lig_T4_1 and methyltransferase domain did not find any similarity (Table 3).

BIR was detected in four bivalve herpesvirus genes and in two AbHV-1-AUS genes (absent in AbHV-1-TAI). Moreover, BIR was highly represented in the *C. gigas* genome, with the oyster protein EKC36433 (initial part of it) showing surprising similarity to one viral gene. Among the viral FICs, only those from dsDNA viruses (not from phages) showed strong conservation of the nine-residue signature of the FIC motif (HPFX(D/E)GNGR) (Roy and Cherfils, 2015), whereas the other hits displayed less-conserved motifs, as observed for some bacterial proteins (Khater and Mohanty, 2015). Some other domains of *Malacoherpesviridae*–specific genes are also present in other dsDNA viruses or in bacteria. The Exo5 exonuclease of bivalve *Malacoherpesviridae* is unique among viruses, whereas the PDDEXK_1 exonuclease of gastropod *Malacoherpesviridae* shows a wide distribution in the *Caudovirales* family and in other DNA viruses. Finally, the *methyltransferase-like* protein unique to AbHV-1-AUS showed similarity with bacterial and (a few) viral proteins (mainly from phages).

### Only *C. gigas* RNA-seq samples include genuine *Malacoherpesviridae* reads

We considered a total of 96 *C. gigas* and 159 *Haliotis* spp. RNA-seq samples, accounting for more than 5.5 billion reads, to identify reads generated by transcriptionally active *Malacoherpesviridae*. Before mapping the mollusk RNA sequences on *Malacoviridae* genomes, we exploited the *C. gigas* genome to filter out all host reads from the *C. gigas* RNA-seq samples. Since a *Haliotid* genome draft was not available, we directly mapped abalone RNA-seq reads on *Malacoherpesviridae* genomes. Stringent mapping of oyster-genome-unmapped reads on known *Malacoherpesviridae* genomes allowed for the selection of 5.483 M reads from the 2.27 billion starting dataset (0.24%), whereas stringent mapping of the *Haliotis* spp. reads produced only 0.01% of mapped reads, with very few reads correctly paired. Manual examination of the mapping files revealed the matching of the gastropod reads to viral oligo-nucleotidic stretches, a fact greatly impairing the mapping specificity. Conversely, the viral reads retrieved from *C. gigas* samples showed a pairing correctness of 94.2%, thus demonstrating that only the *C. gigas* RNA-seq samples contained genuine *Malacoherpesviridae* reads. All analyzed oyster RNA samples (ten of them comprising 90% of the total reads) included at least a few bivalve *Malacoherpesviridae* transcripts (Supplementary File 1). The RNA-seq sample richest in viral reads (3.5 % of the total reads) was from a naturally-occurring infection previously described (Rosani et al., 2015). Viral transcripts were also abundant in samples from more susceptible life stages (larvae, spat, juveniles) and at 24 h post-infection with the variant μVar (only two of the ten richest RNA-seq samples derived from infection trials) (He et al., 2015).

### *Malacoherpesviridae* reads are produced by slightly different viral variants

We further considered the *Malacoherpesviridae* reads mapping on the virus-specific regions previously identified by whole genome alignment (Supplementary File 2). Stringent back-mapping of these reads on sequential combinations of bivalve *Malacoherpesviridae* genomes (0.9 of sequence similarity computed along the whole read length) supported the assignment of 4.903 M (out of 5.483 M reads) to *Malacoherpesviridae* genomes, with residual ~580 k reads failing the genome assignment step (Table 4). Many of the latter (348 k reads) could be mapped anyway by gradually lowering the similarity mapping parameter to 0.5, a fact suggesting a certain degree of variability of viral RNA reads. Sequential genome mapping showed that 20,251 reads not matching an OsHV-1 sequence region could be re-assigned to a genomic region of AVNV, or OsHV-1-SB (60–63 kb) including a 2.7 kb insertion typical of these two genomes. *De-novo* assembling of these 20,251 reads confirmed the presence of ORF125 and ORF126, out of the three AVNV ORFs annotated in this region (ORF125-ORF127). A total of 4,461 other reads were attributed to a deletion on ORF103, typical of both OsHV-1 and OsHV-1-SB but absent in AVNV, suggesting the occurrence of a mixture of viral genotypes in the analyzed RNA-seq samples (Table 5). To further endorse this hypothesis, we searched for viral spliced reads. Spliced reads normally result from the mapping of one mRNA read over an intron sequence, whereas in our analysis they were attributed to insertions or deletions occurring in the viral reference genome. Using ultra confident mapping parameters, like 0.95 and 0.95 for similarity and length fraction, we were able to re-assign a portion of the 11,947 spliced reads to the μVar variant and AVNV genomes (Table 6). All the AVNV-re-assigned reads mapped on a 9-nt deletion of ORF104, the one discriminating AVNV from other *Malacoherpesviridae*.

**Table 4:** Identification of *Malacoherpesviridae* reads in *C. gigas* RNA-seq data.

**Analysis step**	**No. of reads**
Total analyzed reads	2.27 G
Putative *Malacoherpesviridae* reads	5,483,402
*Malacoherpesviridae* assigned reads	4,903,646
Un-assigned reads	579,576
Spliced reads	11,947

*The spliced reads that were recovered with a large gap mapping tool were then re-assigned to Malacoherpesviridae genomes (see Table 6)*.

**Table 5:** Detailed analysis of three RNA-seq samples rich in viral reads.

**Sample ID**	**Total reads**	***C. gigas* unmapped reads [%]**	***Malacoherpesviridae*** **reads**					
			**Total reads**	**Reads on unique regions**	**OsHV-1**	**OsHV-1-μvar**	**AVNV**	**OsHV-1-SB**
E-MTAB-2552	85,335,256	32	3,003,873	274,187	3.1%	95.0%	0.5%	1.5%
SRR334249	26,566,768	39	1,175,934	26,401	7.8%	55.0%	17.0%	20.0%
SRR2002949	6,538,514	36	73,212	5,951	0.9%	93.2%	2.3%	2.4%

*From left to right: sample ID, total number of reads, percentage of C. gigas genomic unmapped reads and, for Malacoherpesviridae, the table illustrates the total number and fraction of reads mapped on unique regions of four viral genomes, with the assignment of percentages to each individual virus*.

**Table 6:** Re-assignment of spliced reads to *Malacoherpesviridae* genomes.

**Spliced reads identified in**	**No. of reads**	**Re-assigned to**			
		**OsHV-1**	**OsHV-1 μVAR (%)**	**OsHV-1-SB**	**AVNV (%)**
OsHV-1	4,640	/	6	–	92
OsHV-1 μVAR	4,583	–	/	–	93
OsHV-1-SB	1,959	–	19	/	51
AVNV	542	–	47	–	/

*Origin and number of spliced reads and the re-assignment percentages are reported*.

### Read mapping highlights few annotation inconsistencies in the OsHV-1 genome

**Figure 4:**
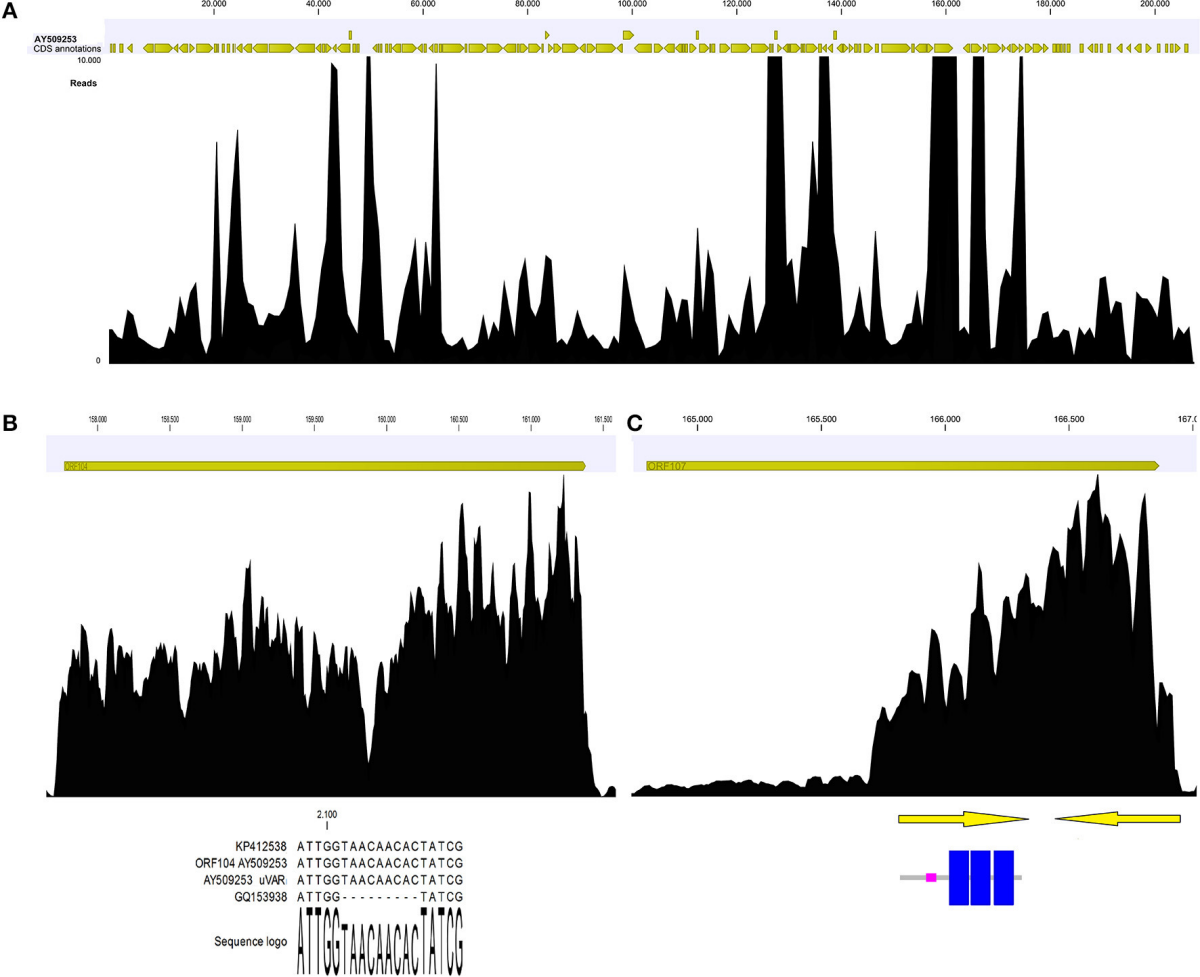
Coverage graph of *Malacoherpesviridae* reads **(A)**. Coverage graph of the whole OsHV-1 genome, as obtained by mapping 4.9 M viral reads with length and similarity mapping parameters set to 0.9. Yellow arrowheads indicate annotated ORFs along the virus genome. Maximum coverage was set to 10,000x. **(B)** Details of the ORF104 coverage graph, with the alignment including the 9-nt deletion causing coverage drop-off. **(C)** Details of ORF107 coverage graph, with two nested ORFs (arrows) that could explain the local coverage bias. One ORF encoded three transmembrane regions (highlighted in blue).

Coverage graph analysis is a powerful tool for detect sequencing anomalies. If reads are unbiasedly produced, the coverage should appear quite homogenous along the entire sequence, even if a 5′ peak may be due to the random fragmentation of multiple mRNA copies of any given transcript, as reported by Arias et al. (2014). Manual inspection of the coverage graph (Figure 4A) along the OsHV-1 genome highlighted a few anomalies. In the ORF104 graph (Figure 4B), two distinct peaks are indicative of a mismatch region impairing the read mapping and confirmative of above reported results. In the ORF107 graph (Figure 4C), highly uneven coverage (from 250x, along the first 1,000 nucleotides, to 4,800x) suggested the presence of two nested ORFs with completely different expression levels. In fact, we recognized two small ORFs, both of 152 AA length and one of them including three transmembrane regions (Figure 4).

Moreover, the coverage graph showed that some reads mapped on the five large intergenic OsHV-1 regions. As previously reported, these regions contain non-coding, disrupted ORFs (Davison et al., 2005; He et al., 2015). Following *de-novo* assembling of the reads corresponding to said genomic regions, we recovered few complete ORFs. Although the assembled reads were retrieved from different RNA samples, nearly all of them clustered in one consensus sequence (Table 7). In detail, the consensus sequences generated for the 50k and 99k regions were similar to ORF88 (transmembrane OsHV-1 protein), whereas the consensus sequence for the 113k region showed similarity to a protein characterized by a domain found only in the crustacean *White spot syndrome virus* (*Nimaviridae*). Finally, for the three consensus sequences generated from the 72k and 94k regions, no similarities to any already annotated sequences were found.

**Table 7:** *De-novo* assembly results of reads mapping on OsHV-1 putative coding regions.

**Genomic region**	**No. of reads**	**No. of samples**	**Assembled contigs**	**Genomic region**	**Identity (*blastp*)**
PUTA_50k	112,855	76	1 (66%)	47,859–49,814	ORF88 [OsHV-1]
PUTA_72k	25,653	81	1 (93%)	73,359–75,186	/
PUTA_94k	17,115	63	2 (100%)	93,041–94,996	/
				95,053–96,949	/
PUTA_99k	55,877	49	1 (100%)	98,220–100,352	ORF88 [OsHV-1]
PUTA_113k	47,072	45	1 (100%)	112,807–114,696	DUF1335-domain containing protein [113,991–114,661]

*Region name, number of extracted reads, and number of samples from which they originated are reported. Number of de novo assembled contigs, genomic region, and blastp annotation are also reported*.

### *Malacoherpesviridae* genomes include few recognizable sequence motifs

We searched known *Malacoherpesviridae* genomes for nucleotidic motifs, oligo-nucleotidic stretches, and PAS. Simple motif searches revealed oligo-nucleotidic stretches (at least eight equal bases, mainly A or T) were preferentially located in intergenic regions (only 20/399 were found in ORFs). Mapping canonical PAS such as AAUAAA and AUUAAA (Arias et al., 2014), we retrieved completely conserved matches in 393 of 559 3′UTRs. Applying a more sophisticated tool (MEME) to the 5′ and 3′ UTR regions, we identified a longer PAS motif in 126 3′UTRs and a second 3′UTR motif. Unfortunately, the diffuse presence of T-stretches in intergenic regions hampered the mapping of reads containing 3′ polyadenylated bases and, hence, an elegant identification of transcript ends, as described by Stern-Ginossar et al. (2012). None of the MEME-proposed 5′-motifs were statistically convincing enough.

### Two DNA recombination-initiating promoter motifs are enriched among *Malacoherpesviridae*

We initially investigated the presence of six HR promoter motifs in two reference *Malacoherpesviridae* genomes (OsHV-1 and AbHV-1-AUS for bivalve and gastropod viruses, respectively) and then we analyzed the resulting data against 2,665 dsDNA viral genomes. Overall, we recognized 1,443,794, and 1,523,194 motifs in original and randomized viral sequences, respectively. As expected, shorter motifs were more present, whereas a classical meiotic recombination motif (CCTCCCCT) (Myers et al., 2005) was found on 1,258 genomes and was labeled as “enriched” only in 88 of them. Complete data are reported in Supplementary File 4. Among the 104 invertebrate dsDNA viruses present in the dataset, only a truncated chi-motif (TGGTGG) (Chuzhanova et al., 2009) was widely enriched (in around 50% of the viruses, including OsHV-1 but not AbHV-1-AUS). The CCTCCCCT motif was computed as being “enriched” only in four invertebrate viruses, namely the two *Malacoherpesviridae, White spot syndrome virus*, and *Invertebrate iridescent virus 6*, a virus for which the interaction with mammalian antiviral systems was recently reported (Ahlers et al., 2016). The distribution of CCTCCCCT motif in known *Malacoherpesviridae* genomes revealed its biased presence, with most them located in the second part genomes.

### Do *Malacoherpesviridae* genomes encode genuine miRNAs?

To infer the presence of miRNAs in *Malacoherpesviridae* genomes, we exploited two *ab-initio* miRNA predictor tools. Compared with VMir, the miRPara algorithm predicted a higher number of putative structured RNAs, with a range of 11–25 predicted miRNAs per genome commonly identified (Table 8). Although most of the *Malacoherpesviridae* genomes are covered by ORFs, only 43–65% of the predicted miRNA structures were located in coding regions, thus indicating their preferential intergenic occurrence. None of the predicted structures found similarity in the miRBase database and, likewise, none of the miRBase hits found a decent match on *Malacoherpesviridae* genomes. Taking into account eight miRNA-seq samples (some of them rich in viral reads, all them belonging to the same oyster batches used to produce developmental RNA-seq libraries), we could not validate any of the predicted viral miRNA regions, nor the miRNA reads mapped to any other viral genome. These analyses do not indicate any genuine *Malacoherpesviridae* miRNA; more focused experiments are needed to definitively clarify this point.

**Table 8:** miRNA prediction results. miRPara and vMir predictions are reported for the six *Malacoherpesviridae* genomes, with total number of predicted regions, common ones and percentage of those located in ORFs.

**Species**	**miRPara**	**vMir**	**common**	**% on ORF**
Total	866	247	96	53
OsHV-1	98	37	11	45
OsHV-1-μVAR	107	37	12	58
OsHV-1-SB	87	43	14	50
AVNV	94	39	14	43
AbHV-1-AUS	242	33	25	52
AbHV-1-TAI	238	58	20	65

### OsHV-1 transcription levels are highly comparable among different RNA samples

**Figure 5:**
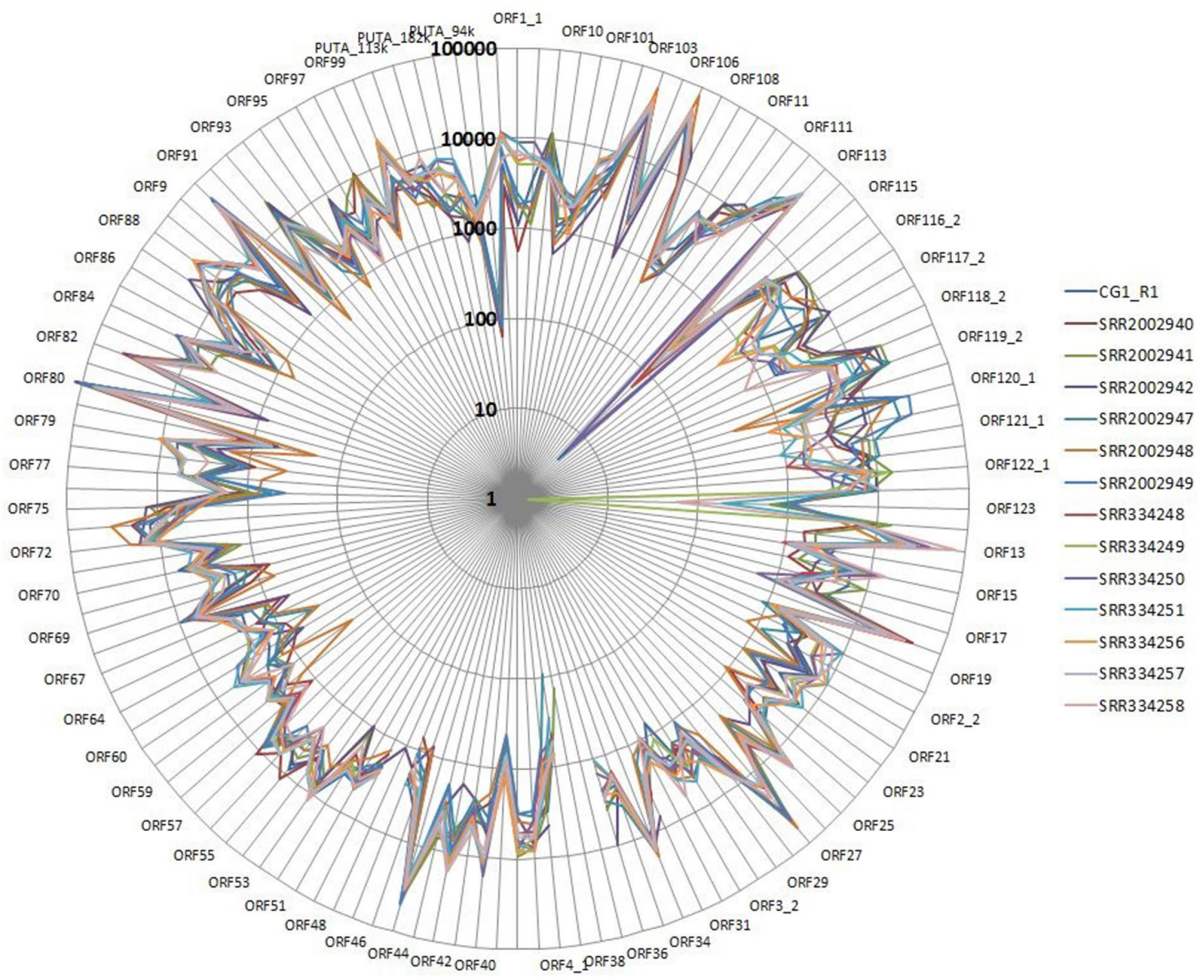
Radar graph depicting the viral TPM values in 14 RNA-seq samples selected as “high”. Expression values are reported with a logarithmic scale.

We performed a detailed expression analysis by mapping the identified *Malacoherpesviridae* reads only on the OsHV-1 reference genome and computing the related expression values in TPM. In addition to the already annotated ORFs, we considered the genomic regions for which we had previously observed a read coverage to be “putative coding regions.” The number of mapped reads as well as non-normalized (nn-TPM) and normalized (TPM) values calculated per ORF are reported in an interactive table offered as an easy tool for interested readers (Supplementary File 5). Although all 96 *C. gigas* RNA-seq samples included viral reads, we classified 14 of them as “high” (i.e., more than 1,000 counted viral reads, Supplementary File 1). As stated above, this classification does not directly correlate to the number of identified *Malacoherpesviridae* reads, since it relies only on the reads that stringently mapped on the OsHV-1 reference genome (and were subsequently counted). The RNA samples in which different viral types contributed to the final amount of *Malacoherpesviridae* reads were remarkable (e.g., SRR334249, Table 5). Digital expression analysis highlighted few expression peaks, namely TPMs > 2M for ORF76, ORF80, ORF29, ORF42, ORF88, and for a putative coding region (PUTA_72k). Principal component analysis (PCA) based on TPM-values clearly supported the “high-expression” grouping based on the counted reads (Supplementary File 6.1). Few ORFs were expressed in almost all RNA-seq samples, such as ORF76, which represents 13% of the total TPMs and has an extremely high relative expression in every sample (although significantly expressed also in “high” samples, as showed by non-TPMs). On the contrary, several ORFs showed a preferential occurrence in selected samples, like ORF27, ORF45, ORF80, ORF82, ORF90, ORF104, ORF107, and ORF113, which grouped in a unique expression cluster (Supplementary File 6.2). Among the several ORFs with unknown function, ORF18 encoding for a 94-aa peptide was highly expressed in multiple RNA-seq samples. As clearly shown in Figure 5, almost all viral ORFs are simultaneously expressed in “high” samples, with very few line interruptions corresponding to ORF36, ORF37, and ORF48 previously reported as not functional (He et al., 2015). In agreement with the results obtained from coverage graph analysis, detectable expression levels were evident for ORF50 (PUTA_72k), ORF62 and ORF63 (PUTA_94k), ORF65 (PUTA_99k), ORF73 (PUTA_113k), and (PUTA_110k).

### SNPs analysis supports the presence of OsHV-1 variants within RNA samples

**Figure 6:**
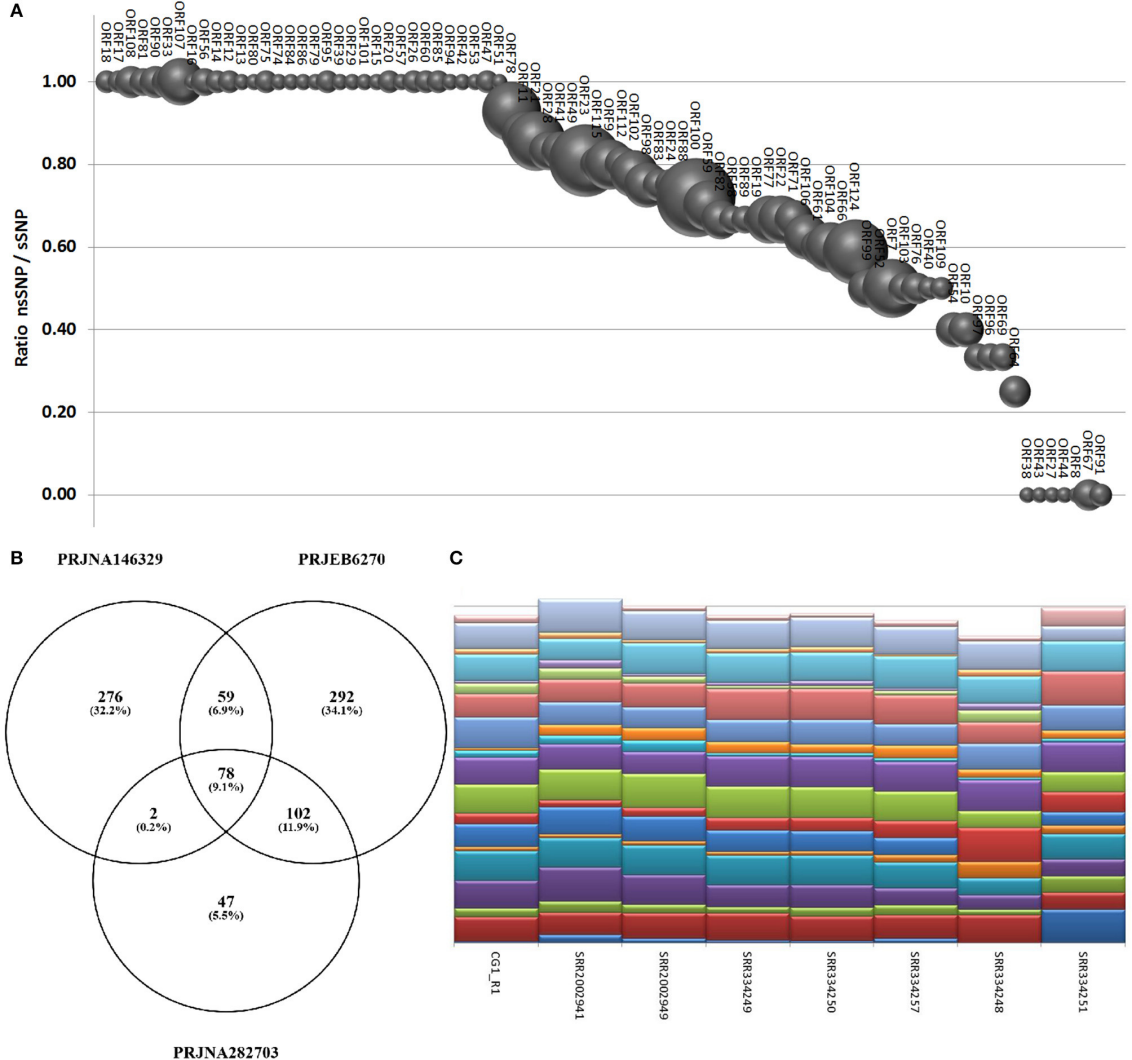
**(A)** SNPs occurrence in OsHV-1 ORFs. The graph depicts the ratio of nsSNP vs. total SNP for the ORFs presenting at least one variation. Globe size is proportional to the number of SNP for each ORF. **(B)** Common and exclusive SNPs indicated in the Venn diagram resulted from the comparison of oyster RNA-seq samples grouped by origin. **(C)** “Genotyping” graph based on 20 polymorphic common OsHV-1 SNPs in the eight samples richest in viral reads (indicated by IDs).

Using a conservative SNP-calling algorithm, we identified 664 variable positions consisting in changes of single nucleotides or small stretches (maximal 5 nt). Eighty-six percent of these SNPs mapped on annotated genes, with the majority of them (75%) involving amino acid substitutions (nsSNP). The total SNPs were distributed on 94 ORFs (40 ORFs are invariant) and non-synonymous (ns) SNP occurred in 87 ORFs (37 ORFs display only nsSNPs, seven only synonymous ones). ORF124 and ORF18 showed the maximal frequency of SNP (1.2%) and of nsSNP (1.1%), respectively (Figure 6A). As reported above, the interactive Supplementary File 5 also includes the SNP frequencies for each OsHV-1 ORF. In spite of their high expression levels, some ORFs showed very few SNPs or appeared invariable, as expected in the case of strict functional constraints. Further analysis of the viral SNPs in the RNA-seq samples grouped by geographical origin produced a core set of 78 common SNPs, with the main part of them supporting an effective difference between sample groups (Figure 6B). Although most of the common SNPs concerned differences between the sampled viruses and the reference OsHV-1 (present at 100% frequency in all groups), 20 SNPs displayed a frequency lower than 95% in at least one group. Using the frequencies of those SNPs to “genotype” undefined OsHV-1-mixtures in eight selected RNA-seq samples, we were able to infer the variable presence of slightly different viruses in single stocks of virus-infected *C. gigas* (Figure 6C), as previously suggested by read mapping on unique genomic regions (reported in Table 5).

## Discussion

HTS methodologies have extraordinarily contributed to the understanding of the viral world in the ocean (Brum et al., 2015). Even though viruses are massively present in coastal waters (Suttle, 2007), only a minimal number of them are represented in sequence databases. This bias allows for the discovery of new viruses in almost every virome study, as recently reported for six new bivalve-associated RNA viruses (Rosani and Gerdol, 2016) and for RNA viruses associated with invertebrates (Shi et al., 2016). Nevertheless, bivalve viromes essentially remain unknown, except for a few pathogenic viruses (Arzul et al., 2017).

In the present work, we focused on the family of *Malacoherpesviridae*, a case study considering their enigmatic evolutionary origin and limited transcriptional/genomic data. Moreover, *Malacoherpesviridae* represent an urgent problem for mollusk aquaculture worldwide: their recurrent association with host mortality outbreaks may lead to a better understanding of their life cycles and dynamic host-pathogen interactions, ultimately for the development of effective prevention and mitigation strategies (Davison et al., 2005; Corbeil et al., 2016; Pernet et al., 2016). The previously reported divergence of *Malacoherpesviridae* from other *Herpesvirales* suggests that long-lasting evolutionary processes may have given rise to the only *Herpesvirales* genomes known to infect invertebrates. Although TEM imaging of *herpes-like* particles supports the occurrence of *Herpesvirales* in corals, recent sequencing data are somewhat elusive (Correa et al., 2016). Moreover, further studies are necessary to assess the presence of *herpes-like* viruses in crustaceans (Bang, 1971; Ryazanova et al., 2015). Intriguingly, we reported Herpesvirales-like sequence elements in genomic scaffolds of *C. teleta* and *Branciostoma* spp. In agreement with Savin and colleagues (Savin et al., 2010) demonstrated that *B. floridae* sequence is highly similar to Abalone *Malacoherpesviridae*), we have shown that *C. teleta*-encoded DNA polymerase is more similar to bivalve *Malacoherpesviridae*, whereas both Branchiostoma-derived DNA polymerase clustered as an *Malacoherpesviridae* outgroup. Taken together, these findings suggest a broader presence of invertebrate-infecting *Herpesvirales*, representing former or extant (still undisclosed) viruses. Although the integration of genetic elements of large DNA viruses in invertebrate host genomes has already been reported (Drezen et al., 2017), further experimental validation is needed to definitively prove the integration of these sequences in host genomes.

The few predictable protein domains of *Malacoherpesviridae* (most of the *Malacoherpesviridae* genes are unique) suggested complex evolutionary paths, including gene transfer events from other dsDNA viruses (in particular, invertebrate viruses), bacteria, and also from mollusk hosts. Among other protein domains, we focused on BIR-containing proteins (putative inhibitors of apoptosis), because apoptotic responses are reported as one of the main bivalve countermeasures to infections due to the μVar variant (Segarra et al., 2014b; He et al., 2015; Martenot et al., 2017) and BIR was found in several dsDNA and ssRNA viruses of invertebrates. Phylogenetic analysis of metazoan and viral BIRs did not indicate any robust evolutionary relationships, thus supporting the hypothesis of extensive gene transfer. We highlighted other common features of invertebrate dsDNA viruses by analyzing promoter sequence motifs involved in virus-protective DNA repair. Invertebrate dsDNA viruses analyzed in this work mainly encode one of the six searched HR promoter motifs, namely a truncated version of the chi-element, whereas almost only *Malacoherpesviridae* are enriched in a classical meiotic recombination motif. These results support the interaction of *Malacoherpesviridae* with host recombination machinery, although probably with promoter motifs that are partially different from the vertebrate ones (and unknown). Commonalities emerged between *Malacoherpesviridae* and *Whispovirus* (*White spot syndrome virus 1*), namely shared domains and similar HR promoter enrichment patterns. An overlap between arthropod (in this case crustaceans) and bivalve viromes was recently reported for several RNA viral families (Shi et al., 2016) which might be a fascinating matter for future studies. Moreover, the reduced species-specificity for *Malacoherpesviridae* somewhat recalls the lack of virus-host co-divergence observed in invertebrate RNA viruses (Shi et al., 2016), and calls for a broad, not species-specific mechanism of action.

Given the lack of permissive cell cultures, we used oyster RNA-seq samples as an effective (and unique) source of viral reads. In the so-called “RNA-seq dark matter” (Ponting and Belgard, 2010), the accidental or deliberate sequencing of OsHV-1-infected oysters can make millions of viral reads available, revealing active viral transcription. Despite the analysis of numerous *Haliotid* RNA-seq samples, no similar results could be achieved for abalones. Read mapping on *Malacoherpesviridae* unique genome regions as well as SNP analysis suggested the presence of more than one viral variant within and between RNA-seq samples. One virus type was preferentially present (i.e., more transcriptional active) in each host transcriptome sample, although a single virus encoding all the variants typical of different mollusk viruses might exist. Advanced ultra-deep RNA and DNA sequencing would be optimal to ascertain this point.

Although OsHV-1 was apparently suppressed in many of the analyzed samples (e.g., samples with less than a thousand viral reads), only proper controls could validate this hypothesis and assign the expressed viral ORFs to the persistent virus phase. Therefore, we only observed the broad expression of ORF76 in almost all samples with few viral reads. Interestingly, a limited structural similarity of ORF76 with the human nucleoporin (data not shown) postulates a role of this protein in the viral entry inside cell nucleus. Viral expression profiles were particularly informative in highly infected samples (supposed lithic virus phase), with most viral ORFs actively expressed and no predominant expression of single ORFs. Actually, the concordance between ORF expression ratios in these samples was a remarkable finding possibly revealing the functional importance of many viral proteins during virus replication, like in the case of the highly expressed dUTPase (ORF27, completely lacking nsSNPs) in agreement with (Segarra et al., 2014a,b). Functional validation based on *in-situ* hybridization, western blot and recombinant proteins is definitively needed to investigate the functional role of viral ORFs, as previously initiated (Martenot et al., 2016, 2017; Segarra et al., 2016).

Despite the lack of functional data for *Malacoherpesviridae*, conserved intergenic features might reveal hidden traits of virus-host co-existence mechanisms. The current expansion of mollusk genomics is expected to answer some of the open questions mentioned in this work. Our analyses demonstrated that viral transcriptomics may greatly contribute to the understanding of the molecular facets of new viral variants including ORFs and aminoacidic changes crucially related to the pathogenic nature of certain variants, and may be useful to improve diagnostic qPCR-based methods.

## Author contributions

UR and PV designed the analytical pipeline, UR performed the bioinformatic analysis, UR and PV prepared the manuscript.

### Conflict of interest statement

The authors declare that the research was conducted in the absence of any commercial or financial relationships that could be construed as a potential conflict of interest.

## Supplementary material

Supplementary File 1 Sequencing samples considered in the present work. Experiment description, SRA ID and sample description, number of millions reads, and number of identified *Malacoherpesviridae* reads are reported. Underlined samples with more than 1,000 viral reads are indicated as “high” samples.

Supplementary File 2 Whole genome alignment of five *Malacoherpesviridae* genomes using progressive MAUVE. From top to bottom: OsHV-1, OsHV-1-SB, AVNV, AbHV-1-AUS, and AbHV-1-TAI. Conserved sequence blocks are reported in the same color whereas the available annotations are reported as empty boxes just below the colored blocks for each genome.

Supplementary File 3 PFAM domains encoded by *Herpesvirales*. Domain name, PFAM accession, domain description, and length are reported. Each domain is labeled (“yes”) if present in bivalve *Malacoherpesviridae*, gastropod *Malacoherpesviridae*, or other *Herpesvirales. Malacoherpesviridae*–exclusive domains are highlighted in yellow.

Supplementary File 4 Distribution of six HR promoters in 2,665 dsDNA viral genomes. From left to right: virus name, NCBI accession, genome length, number of predicted proteins, and main host and, for the six recombination-initiating motifs, the table illustrates their quantity, their genome-length normalized frequencies, and their enrichment ratios.

Supplementary File 5 Interactive visualization of the expression patterns of viral ORFs in *C. gigas* RNA-seq samples. Number of mapped reads per sample, non-normalized, and TPM expression values as well as total and non-synonymous SNPs are reported for any selected ORF.

Supplementary File 6 ORF expression analysis. Figure SF6_1. Correlation plot of Principal Component Analysis (PCA). Red and green dots indicate RNA-seq samples labeled as “high” or not labelled, respectively. Figure SF6_2. Clustering of viral ORFs. ORFs are clustered based on their TPM values using an Euclidean distance algorithm. “High” samples are framed in black. See the color legend at the bottom of the figure.
