# Supplementary figures and images for: Oyster RNA-seq Data Support the Development of Malacoherpesviridae Genomics
Source: Front Microbiol. 2017 Aug 9;8:1515. doi: 10.3389/fmicb.2017.01515 (PMC5552708; doi:10.3389/fmicb.2017.01515)

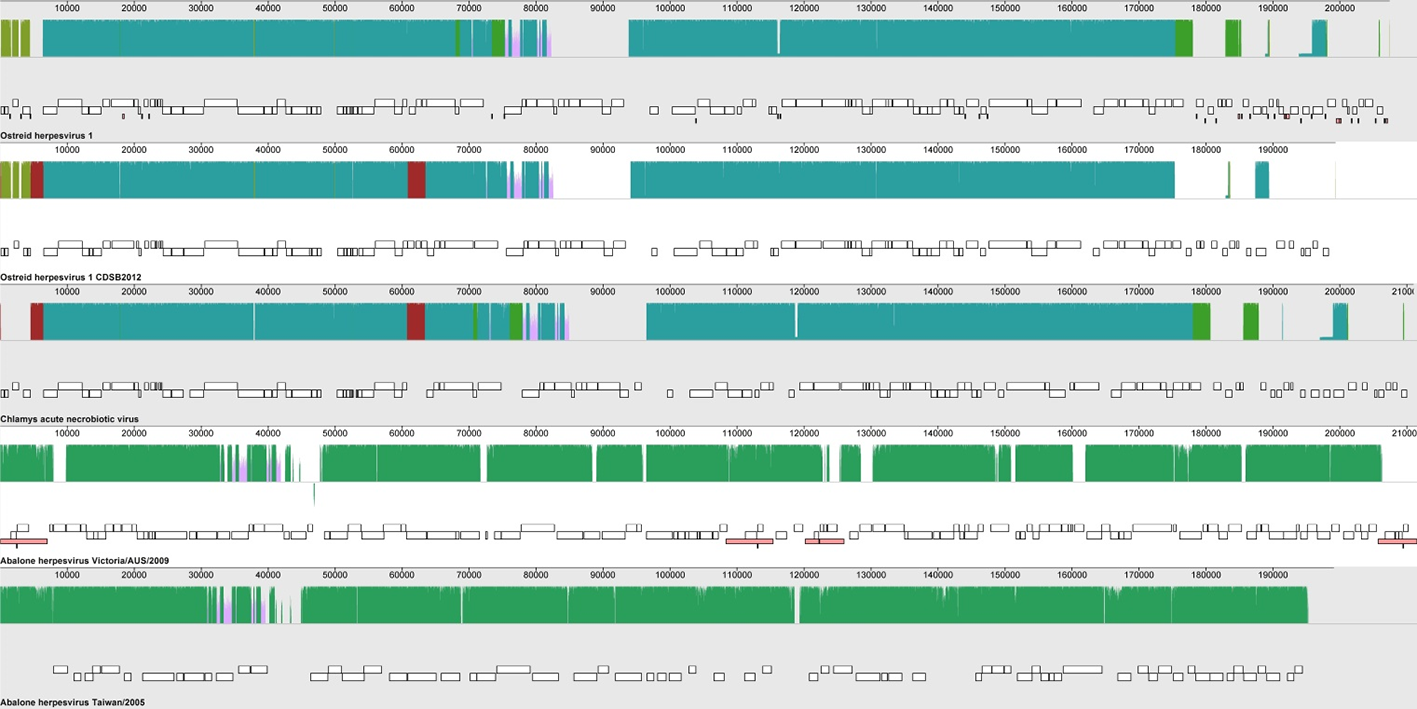

Supplement: Supplementary File 2 — Whole genome alignment of five Malacoherpesviridae genomes using progressive MAUVE. From top to bottom: OsHV-1, OsHV-1-SB, AVNV, AbHV-1-AUS, and AbHV-1-TAI. Conserved sequence blocks are reported in the same color whereas the available annotations are reported as empty boxes just below the colored blocks for each genome. [file Image1.TIF]
