# Supplementary material for: Oyster RNA-seq Data Support the Development of Malacoherpesviridae Genomics
Source: Front Microbiol. 2017 Aug 9;8:1515. doi: 10.3389/fmicb.2017.01515 (PMC5552708; doi:10.3389/fmicb.2017.01515)

**Supplementary File 6**. ORF expression analysis. **Figure SF6_1.** Correlation plot of Principal Component Analysis (PCA). Red and green dots indicate RNA-seq samples labelled as ‘high’ or not labelled, respectively


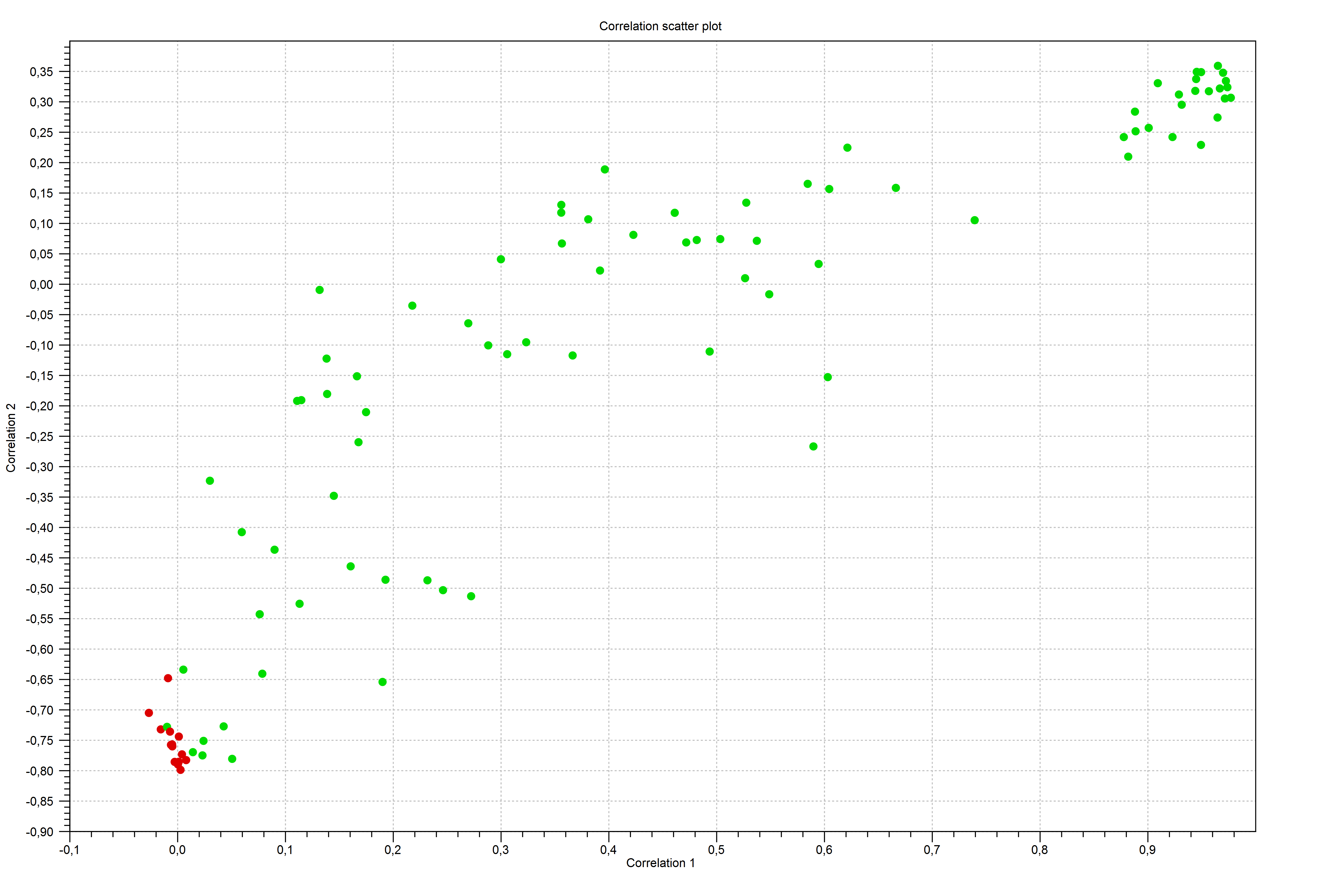


. **Figure SF6_2**. Clustering of viral ORFs. ORFs are clustered based on their TPM values using an Euclidean distance algorithm. ‘High’ samples are framed in black. See the colour legend at the bottom of the figure.


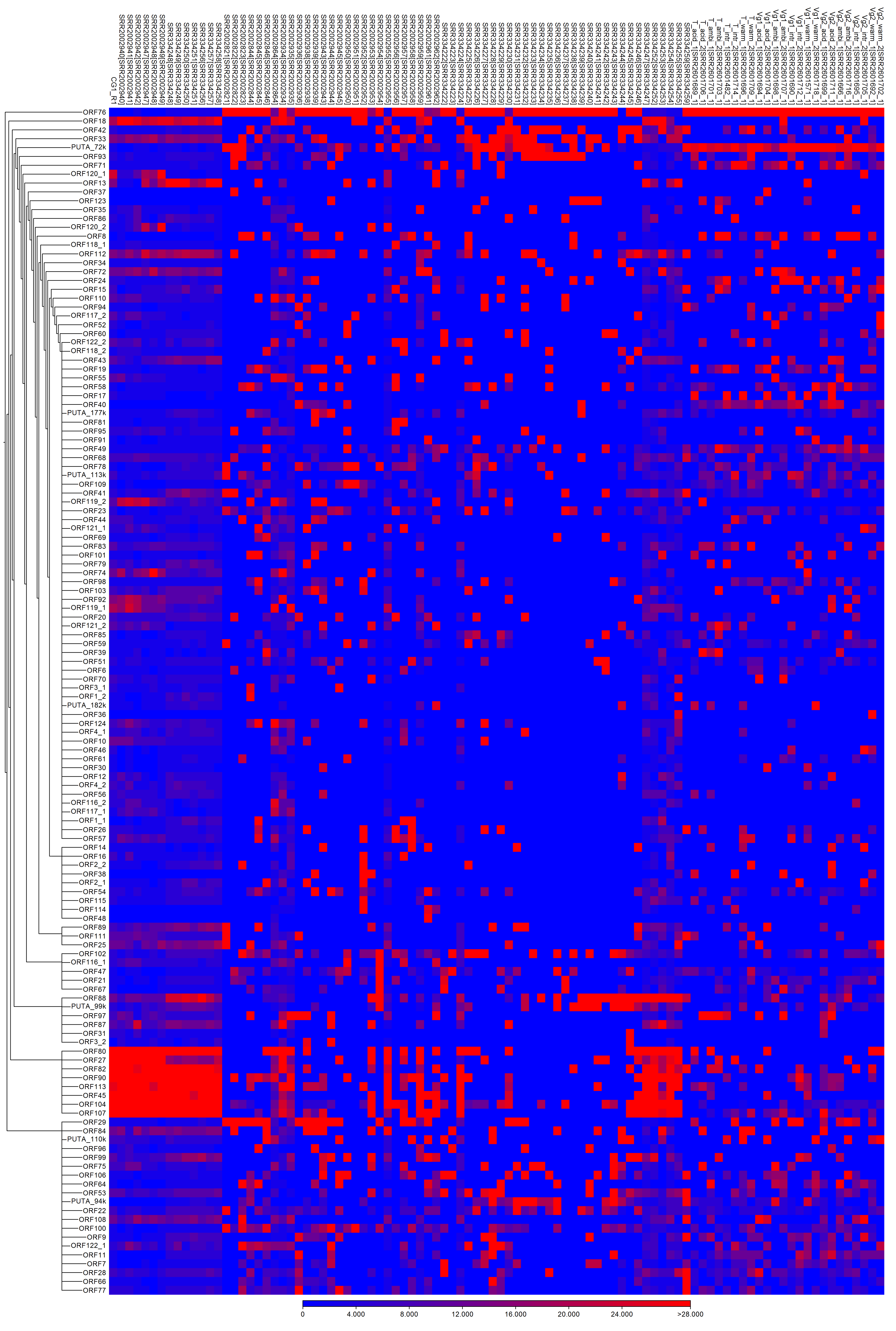

Supplement: Supplementary File 6 — ORF expression analysis. Figure SF6_1. Correlation plot of Principal Component Analysis (PCA). Red and green dots indicate RNA-seq samples labeled as “high” or not labelled, respectively. Figure SF6_2. Clustering of viral ORFs. ORFs are clustered based on their TPM values using an Euclidean distance algorithm. “High” samples are framed in black. See the color legend at the bottom of the figure. [file Table6.DOCX]
